# Persistent Spinal Pain Syndrome: A Study of Contact Heat-Evoked Potentials

**DOI:** 10.7759/cureus.80174

**Published:** 2025-03-06

**Authors:** Bruno Lima Pessôa, Eduardo Davidovich, Osvaldo Nascimento, Wilhelmina N Hauwanga, Billy McBenedict

**Affiliations:** 1 Neurosurgery, Fluminense Federal University, Niterói, BRA; 2 Neurology, Fluminense Federal University, Niterói, BRA; 3 Cardiology, Gaffrée and Guinle University Hospital, Federal University of the State of Rio de Janeiro, Rio de Janeiro, BRA

**Keywords:** back pain, central sensitization, contact heat evoked potentials, neuropathic pain, persistent spinal pain syndrome

## Abstract

Introduction: Persistent spinal pain syndrome (PSPS) type 2 is a chronic condition characterized by low back pain, with or without radiating limb pain, persisting after spinal surgery. The underlying pathophysiology remains unclear, with potential contributions from altered central pain processing mechanisms.

Objective: This study investigates the role of central sensitization in PSPS using contact heat-evoked potentials (CHEPs), an electrophysiological tool for assessing spinothalamic tract integrity and central pain processing.

Materials and methods: The study included 36 healthy controls and 15 PSPS patients with neuropathic pain (NP) localized to the L4, L5, and S1 dermatomes, stimulated at the L1 dermatome. CHEP parameters, including N2-P2 amplitude, N2 latency, and P2 latency, were compared between groups.

Results: Significant differences were identified, with PSPS patients demonstrating prolonged N2 and P2 latencies (p=0.008 and p=0.005, respectively) and reduced N2-P2 amplitude (p=0.025). Receiver-operating characteristic (ROC) analyses revealed N2 latency as the most reliable diagnostic parameter (AUC=0.81, sensitivity 67%, specificity 80%). Approximately 68% of PSPS patients exhibited abnormal CHEP values, suggesting spinothalamic tract dysfunction.

Conclusions: These findings support the hypothesis of altered central pain processing in PSPS, consistent with previous studies on NP and central sensitization. However, no statistically significant correlation was observed between pain intensity (verbal rating scale) and CHEP parameters (p=0.06), potentially due to the limited sample size. CHEP is a valuable non-invasive tool for exploring central pain mechanisms in PSPS with potential diagnostic and therapeutic implications. Limitations include the small sample size and potential confounders, such as subclinical polyneuropathy in controls. Further research is recommended to complement these findings and explore the broader implications of central sensitization in PSPS and other chronic pain conditions.

## Introduction

Over the past decades, spinal surgeries, particularly lumbar fusion (arthrodesis), have significantly increased [1]. While the success rate of lumbar spinal surgery can exceed 80% in some studies, persistent back pain remains a concern for many patients following lumbar discectomy and arthrodesis [2,3]. In 2019, the International Association for the Study of Pain updated its chronic pain classification, replacing the term "failed back surgery syndrome" with "chronic pain after spinal surgery" [4]. Subsequently, experts introduced the term "persistent spinal pain syndrome" (PSPS), which is further classified into type 1 (pain without prior surgery) and type 2 (pain following surgery) [5]. This article focuses on patients with PSPS type 2.

PSPS lacks objective diagnostic criteria but is generally defined as chronic low back pain, with or without lower limb pain, persisting after one or more spinal surgeries [6,7]. PSPS is more commonly observed in patients with a history of spinal surgery for lumbar disc herniation or lateral recess stenosis [6]. Notably, routine neurophysiological tests, such as electroneuromyography and somatosensory-evoked potentials, may yield normal results if the spinothalamic tract is exclusively affected [8,9]. Moreover, there is no clear correlation between imaging findings and the severity of pain in PSPS. Given that other pain conditions can result from heightened central pain processing [10,11], an important question arises: Could a central phenomenon contribute to the clinical presentation in PSPS patients? Persistent activation of dorsal horn neurons may lead to increased neuronal firing, culminating in central sensitization [12,13].

Contact heat-evoked potentials (CHEPs) have emerged as a valuable electrophysiological tool in pain research [14-16]. More recently, CHEPs have also been utilized to assess the spinothalamic tract in cases of spinal cord lesions [15,17,18]. Applying heat stimulation, CHEPs elicit brain potentials recorded using an electroencephalogram. Heat stimuli are delivered at 70°C per second, reaching a peak temperature 360 ms after stimulation onset. This repetitive and wide-area excitation of cutaneous nociceptors is achieved by releasing heat stimuli [14]. Three peaks (Cz/N550, Cz/P750, and Pz/P1000) can be individualized following the CHEP stimuli. The late Cz/N550 (N2) component represents A-delta fiber, and the very late Pz/N1000 (P2) element at 800-1000 ms represents C-fiber stimulation [14,19]. Consequently, a signal is conducted through C- and A-delta fibers to the central nervous system, permitting a real evaluation of thermal nociceptive pathways [14,20]. Therefore, CHEPs can be regarded as a reliable electrophysiological method available to evaluate the central processing of pain [21].

Importantly, few electrophysiology studies have emphasized the neuropathic component of PSPS [9,22-24]. While many papers have highlighted the role of CHEPs in studying small fiber injury and spinal cord lesions [15,25-28], no CHEP study has yet focused on central pain processing in PSPS. Therefore, using such a method could provide a better understanding of the pathophysiology of PSPS and, consequently, improve its treatment. We tested the central sensitization hypothesis by studying patients with PSPS and NP from the L4, L5, and S1 dermatomes and stimulating at L1.

To facilitate early access to the data, this article was previously posted on the Research Square preprint server on May 10, 2024 (https://doi.org/10.21203/rs.3.rs-4326398/v1).

## Materials and methods

Controls

We analyzed 36 healthy volunteers aged between 18 and 75 years with normal neurological examinations. There were 18 men (aged 47.82 ± 17.01 years, range 28-78 years) and 18 women (aged 49.22 ± 16.78 years, range 27-75 years). The age between both genders was not significantly different. No previous history of drug use, medications, or diseases predisposing to polyneuropathies, such as diabetes, amyloidosis, sarcoidosis, hepatitis, kidney failure, or alcoholism, was found. All data were collected retrospectively. Normative data were obtained from the healthy participants and utilized for comparison purposes (data not shown). All participants, including those in the control group, provided consent in accordance with the Helsinki Convention. The Neuroscience Center at Federal Fluminense University approved the study under the number 03476212.0.0000.5243.

Patients

All 17 patients experienced low back and lower limb pain. They were diagnosed with neuropathic pain (NP) using the DN4 questionnaire (with a positive score) and neurological examination (standard neurological examination, with particular attention to evaluating small fibers). The neurological exam included cranial nerve assessment, muscle strength evaluation, assessment of muscular atrophy, sensory function testing at lumbar dermatomes (using the pinprick test), and evaluation of deep reflexes.

Contact heat stimulation and recordings

All patients were positioned laterally to assess the lumbar region during the examination. The room maintained an average temperature of 22°C to ensure patient comfort. They were instructed to remain awake throughout the test, refrain from blinking, and concentrate on a specific imaginary point predetermined by the examiner. The maximum temperature reached during the stimulation was 51° Celsius to standardise and prevent cutaneous lesions. Using thermal stimulator contact (Medoc Ltd., Ramat Yishai, Israel), the lumbar spine (L1, spinous process) was stimulated. Each stimulus was made in a pulsatile fashion, lasting up to 12 seconds, moving the thermode randomly around L1 (and, therefore, in different locations). Doing so, we avoided the process of habituation of the painful phenomenon. The same gradation pressure with the thermode on the skin was attempted during the examination for each stimulus.

Following each stimulus, we instructed the patients to rate the pain elicited by the CHEPs using the verbal rating scale (VRS), where 0 indicated no pain and 10 represented the worst possible pain. The stimulation parameters used were an intensity of 51°C, an interval between stimuli (ISI) of 8-12 seconds, and two stimulus series (10 stimuli each) carried out at L1, randomly stimulating within the same dermatome. There was at least a 30-second interval between two subsequent series, and the thermode was subtly moved horizontally after each stimulus to avoid overlapping between two subsequent stimulation sites. An experienced neurophysiologist executed CHEPs and remained blinded to all the exams.

Statistical analyses

The study involved conducting between-group analyses and comparing a control group with PSPS patients. N2 latency, P2 latency, and N2-P2 amplitude were considered as dependent variables. A p-value <0.05 was considered statistically significant. All the numerical data were revealed by mean ± SD. The Mann-Whitney test was used to analyze non-parametric data. An independent t-test compared the different CHEP variables between the control group and the PSPS one for univariate analyses and MANOVA for the multivariate ones. In this regard, a p-value was considered statistically significant when <0.02 (0.5 divided by 3, the number of variables) when MANOVA was statistically significant, to elucidate the possible influence of the groups on CHEP variables. Using normative data obtained from healthy participants (data not shown), we calculated the percentage of altered latencies and amplitudes for the PSPS group.

The neurological examination, particularly the pinprick test, was considered the gold standard for diagnosing spinothalamic impairment and determining the presence of neuropathic pain, along with the DN4 questionnaire. Logistic regression analysis and area under the ROC curve calculation were employed to assess the accuracy of CHEPs in diagnosing spinothalamic damage. This helped determine the sensitivity and specificity of each CHEP parameter (N2 latency, P2 latency, or N2-P2 amplitude) in differentiating between the control and PSPS groups. Additionally, the areas under the curve were used to evaluate discrimination: values ≤0.5 indicated the absence of discrimination, 0.7-0.8 suggested acceptable discrimination, 0.8-0.9 indicated excellent discrimination, and values ≥0.9 were considered exceptional. Furthermore, Spearman’s rank correlation tests were used to analyze the correlation between VRS and N2-P2 amplitude. Statistical analyses were performed using SPSS Statistics version 20 (IBM Corp. Released 2011. IBM SPSS Statistics for Windows, Version 20.0. Armonk, NY: IBM Corp.).

## Results

Differences between the groups

Thirty-six normal participants were initially enrolled for the study, comprising 18 females and 18 males. However, one subject declined to participate, resulting in a total of 17 women and 18 men remaining in the study. Additionally, two out of the 17 patients initially diagnosed with PSPS were excluded due to a subsequent diagnosis of polyneuropathy, leaving 15 patients in the PSPS group (Table 1).

**Table 1:** Demographics and CHEPS data of PSPS patients and controls Continuous variables are presented as mean (SD). There was no difference in epidemiological variables and VRS after a stimulus between the groups. No correlation was uncovered between spontaneous VRS, DN4, duration of pain, type of surgery, the presence of radiculopathy, MRI findings, and CHEP parameters (data not shown). CHEP data differed between the control and PSPS groups. * Chi-square, Student’s t-test, or Mann-Whitney, ** provoked by CHEP thermode, *** through electroneuromyography NA: not applicable, PSPS: persistent spinal pain syndrome, SD: standard deviation, VRS: verbal rating scale, CHEPS: contact heat-evoked potentials, M: male, F: female, MRI: magnetic resonance imaging

	PSPS (n=15)	Controls (n=35)	p-value*
Age (mean; SD)	51.76 (8.9)	48.54 (16.6)	0.461
Gender (M/F)	9/6	18/17	0.514
Height (mean cm; SD)	168.80 (7.3)	168.00 (9.5)	0.7
VRS (median) after stimulus	4	5	0.4
Spontaneous VRS (median)	6	NA	-
Dn4 (median)	5	NA	-
Duration of pain, months (mean)	11	NA	-
Type of surgery			
Laminectomy	11/15	NA	-
Arthrodesis	4/15	NA	-
Radiculopathy***	8/15	NA	-
MRI findings			
Arachnoiditis	3/15	NA	-
Epidural fibrosis	5/15	NA	-
Both	3/15	NA	-
Recordable CHEPs	10/15	34/35	0.001
N2-P2 amplitude (mean uV (SD), range)	17.1 (7.24), 7.3-32.6	25.61 (11.6), 5.7-66.8	0.025
P2 latency (mean ms (SD), range)	628.3 (135.1), 506-1000	568.6 (80.3), 458-950	0.04
N2 latency (mean ms (SD), range)	489 (117.6), 394-824	424.6 (64.4), 344-732	0.008

The mean age did not significantly differ between the control and PSPS groups (48.54 and 51.76, respectively; p=0.46). Similarly, there was no significant difference in the proportional gender distribution, with nine males and six females in the PSPS group and 18 men and 17 women in the control group (p=0.5). The mean height was also comparable between the two groups (168 cm for the control group and 168.8 cm for the PSPS group; p=0.7). Moreover, the median VRS was 5 for the control group and 4 for the PSPS group (p=0.4), while the median DN4 scale was 5 for PSPS patients. Regarding the CHEP parameters, there were significant differences between the groups regarding N2-P2 amplitudes, N2 latency, and P2 latency (p=0.025, p=0.008, and p=0.047, respectively) (Table 1).

MANOVA revealed a significant difference between the control and PSPS groups in the multivariate analysis (p=0.003). However, only the N2 (p=0.008) and P2 (p=0.005) latencies were determinant for this difference, with the amplitude N2-P2 presenting p=0.025, being, therefore, higher than the level of significance adopted (p<0.01). There was no difference in epidemiological variables and VRS after a stimulus between the groups. No correlation was uncovered between spontaneous VRS, DN4, duration of pain, type of surgery, and radiculopathy (Table 1).

PSPS patients

Notably, upon applying the normative data from the control group, abnormal values for latencies and amplitudes were detected in 64% of cases for N2 latency. Furthermore, when at least one parameter from CHEPs (N2 latency, P2 latency, or N2-P2 amplitude) was considered indicative of pain pathway impairment at L1, 68% of patients with PSPS exhibited altered CHEPs, suggesting potential spinothalamic tract impairment.

**Figure 1:**
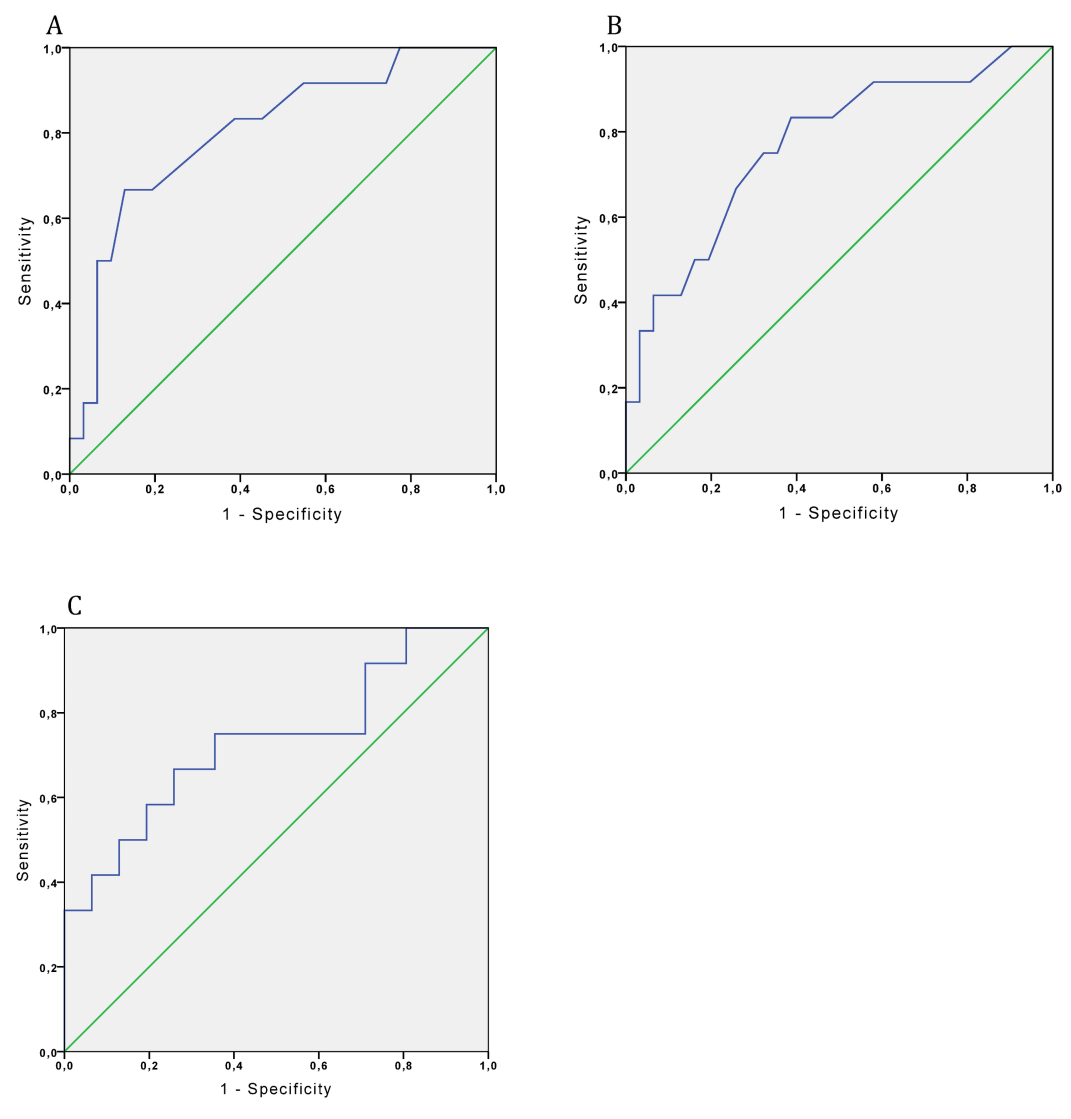
Differentiation between the control and PSPS groups at L1, employing (A) N2 latency ROC-AUC (sensitivity 67%, specificity 80%), (B) P2 latency ROC-AUC (sensitivity 50%, specificity 80%), and (C) N2-P2 amplitude ROC-AUC (sensitivity 50%, specificity 87%) ROC: receiver-operating characteristic, AUC: area under the curve

Considering the cut-off values for latencies and amplitudes obtained through the ROC curve, the parameter that exhibited the largest area under the curve was N2 latency (AUC=0.81, sensitivity 67%, specificity 80%, p=0.002), followed by P2 latency (AUC=0.77, sensitivity 50%, specificity 80%, p=0.007) and N2-P2 amplitude (AUC=0.73, sensitivity 50%, specificity 87%, p=0.02) (Figure 1).

**Figure 2:**
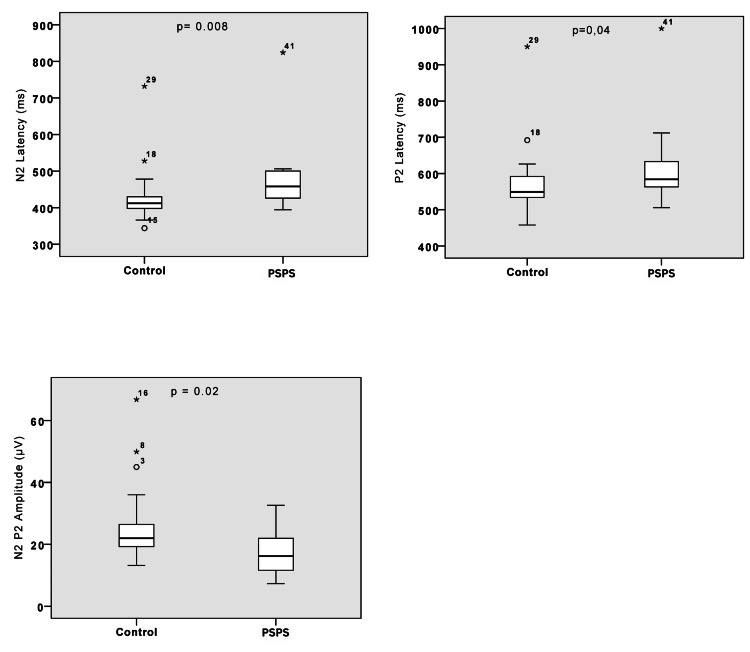
Box plots illustrate the CHEP value differences between the control and PSPS groups PSPS: persistent spinal pain syndrome, CHEPs: contact heat evoked potentials

Most (73%) of patients with PSPS were accurately classified using the variable N2 latency, followed by 54% for P2 latency and 45% for N2-P2 amplitude (Figures 1-2). When only one of the altered CHEP parameters was considered a surrogate for diagnosing pain pathway lesions, CHEPs successfully diagnosed PSPS in 73% of cases (8 out of 11). Among all the parameters measured in CHEPs, the N2-latency was identified as the most significant for distinguishing between the control group and the PSPS group, and discrepancies were observed in the univariate analyses for N2-P2 amplitudes (p=0.025), N2 latency (p=0.008), and P2 latency (p=0.005) (Figure 2).

Correlation between low back pain (VRS) and N2-P2 amplitudes

A non-statistically significant correlation was observed between the variables VRS and N2-P2 amplitudes when considering L1 as the stimulated site in the PSPS group (Spearman’s correlation = 0.3, p=0.06). In terms of CHEP qualitative analyses, recordable CHEPs, we successfully obtained CHEP waveforms at L1 in 34 out of 35 normal participants and 9 out of 15 PSPS patients (x2 (1) = 12, p = 0.001).

## Discussion

The current research identified (1) diminished amplitudes, (2) prolonged latencies, (3) a substantial portion of PSPS patients categorized with abnormal CHEPs, and (4) varying rates of reproducible CHEPs between the control and PSPS cohorts. In recent years, several authors have proposed altered central pain and central sensitization as similar phenomena to explain clinical findings in patients with NP stemming from various causes [13,29]. According to studies on spinal cord injury and NP, the spinothalamic tract lesion is considered a prerequisite for NP development [30,31]. Given that CHEPs are recognized as a functional tool for assessing the integrity of the spinothalamic tract in patients with spinal cord disorders, their utilization would benefit studying NP in patients with PSPS [14,32].

To date, most published series have not documented the utilization of electrophysiological studies in PSPS, with only a few employing QST for NP assessment [23]. Increasingly utilized in examining small fiber lesions, CHEPs have demonstrated utility in diagnosing various pain syndromes [14,15,33,34]. Given the robustness and reproducibility of latency (N2 and P2) and amplitude potentials, CHEPs have seen expanded usage in pain condition studies [14,20,25,35-37]. Furthermore, CHEPs may serve as a predictor of treatment or surgical success [38]. However, to our knowledge, there are no reports in the literature regarding its application in the investigation of PSPS. Notably, only a few studies have investigated the lumbar spine using CHEPs [27,38]. Due to its proximity to the spinal cord, high reproducibility rates of CHEPs in this area may be achieved, allowing for the application of this method in studying painful conditions of the lumbar region [27].

To standardize the epidemiological characteristics between the control and PSPS groups, we considered variables such as gender, age, and height. In this regard, we successfully obtained similar proportions of participants in terms of gender (p=0.5), age (p=0.46), and height (p=0.7). Therefore, comparisons between the groups regarding the CHEP variables were conducted homogeneously.

Importantly, numerous studies have demonstrated reduced amplitudes and prolonged latencies in patients with dysfunction of pain pathways and NP stemming from various causes [11,25,39,40]. Thus, we compared the control group with the PSPS group regarding N2-P2 amplitude, N2 latency, and P2 latency. Consistent with findings in the literature, we observed similar discrepancies in univariate analyses for N2-P2 amplitudes (p=0.025), N2 latency (p=0.008), and P2 latency (p=0.005). Furthermore, MANOVA analyses revealed a significant difference in CHEP parameters between the groups in a multivariate analysis, highlighting their distinct condition. This aligns with previous studies that have identified varying CHEP values when comparing a control group with another group affected by a specific pathology, thereby supporting the role of CHEPs in pain research [25,27,33].

Several studies have addressed the acquisition of normal values and cut-off points to determine whether an individual has altered CHEPs [14,25,41]. Consequently, abnormal values could indicate pain pathway impairment. Utilizing normative CHEP data (data not shown), we identified spinothalamic tract lesions (indicated by CHEP alterations) in 68% of patients. Additionally, N2 latencies demonstrated satisfactory diagnostic accuracy in differentiating between the control and PSPS groups, with 73% of patients correctly classified with pain pathway impairments in PSPS patients during ROC analyses. These results closely resemble those of Parson et al. [27], who found 67% of patients correctly classified with small fiber lesions in NP and type 2 diabetes [27]. Moreover, the same authors obtained an area under the ROC curve of 0.778, with a sensitivity of 67% and specificity of 87% (similar to our findings; AUC=0.81, sensitivity 67%, and specificity 80%) [27]. Consequently, the ROC findings (Figure 2) support the notion that CHEPs are reliable for studying pain phenomena in PSPS. The N2 latency ROC-AUC was greater (0.804, p=0.002) than the AUC for P2 latency (0.769; p=0.007) and N2-P2 amplitude (0.731; p=0.02).

According to several authors, a positive correlation between VRS scores and N2-P2 amplitudes is anticipated [14,27,34,37]. However, we did not observe a statistically significant correlation between these variables (p=0.06). Notably, a higher median VRS was observed in the control group, which could reinforce the integrity of the spinothalamic tract. However, this difference did not reach statistical significance either (p=0.4). A plausible explanation for both phenomena could be the limited size of our sample, which may have hindered us from obtaining significant results in this regard.

As previously noted by other authors, N2 and P2 components are reliably evoked in many individuals [14,25]. When comparing the control group with the PSPS group, a greater percentage of waveforms were produced by CHEPs in the control group (x2 (1) = 12, p=0.001). However, one unresolved question is whether non-reproducible CHEPS should also be considered indicative of altered CHEPs. Theoretically, impairment in the spinothalamic tract could decrease N2-P2 amplitudes, thereby increasing the rate of non-recordable CHEPS [15,18,38,42]. Hence, this issue warrants further investigation in future studies. These findings lead us to hypothesize about the role of spinothalamic tract impairment in PSPS patients and, consequently, altered central pain processing. Since we stimulated at a rostral level (L1), observing a significant number of abnormal CHEPs in patients with NP from L4, L5, and S1 dermatomes, our results confirm those reported in other studies [18,43]. Specifically, our findings support the possibility of distorted processing of somatosensory pathways in dermatomes rostral to the level of injury in patients with spinal disorders [18,43].

In this context, Curatolo et al., in a review of human and animal studies, elucidated alterations in the sensitization of central nociceptive pathways in patients with NP and whiplash injuries [43]. Notably, they illustrated this phenomenon by observing altered quantitative sensory testing (QST) results when stimulating tissue without damage, such as in the altered cervical region when the neurological deficit was primarily in the lower limbs [43].

Furthermore, it is worth mentioning that some studies have also demonstrated central sensitization in idiopathic chronic low back pain (CLBP) [10,44]. In an intriguing study conducted by Giesecke et al., CLBP patients underwent sensory tests and functional MRI scans, revealing increased pain perception and neuronal activation in pain-related cortical areas compared to controls [10]. Similarly, the same authors investigated central pain processing using functional MRI and painful pressure stimuli in CLBP patients and controls [44]. They assessed regional cerebral blood flow (rCBF) in the periaqueductal gray. They found a significantly lower increase in rCBF in this area and a higher increase in rCBF in somatosensory areas in CLBP patients. These findings collectively underscore the impairment of inhibitory pain systems in CLBP patients [44].

Although our study may be considered original in its focus on CHEPs and PSPS, numerous other studies have reported similar CHEP changes when analyzing pain pathway impairment [27,33,45]. Therefore, the results presented here underscore the potential utility of CHEPs for future studies on PSPS, given its reliability and non-invasive nature. However, it is essential to acknowledge some limitations of our study. Firstly, the small sample size of patients described herein restricted our ability to conduct a more robust statistical analysis. Additionally, we must consider whether distractions and attention during the examination may have influenced the number of recordable CHEPs obtained [26]. Furthermore, it is important to note the possibility that some individuals in the "normal" control group may have subclinical small fiber polyneuropathy, as previously suggested by Lagerburg et al. [26]. Consequently, this could lead to decreased amplitudes and increased latencies, potentially affecting our control group analyses [36].

Limitations of the study

While this study provides valuable insights into the role of central sensitization in PSPS type 2 using CHEPs, several limitations should be acknowledged. First, the small sample size of the PSPS patients limits the generalizability of the findings. A larger cohort is necessary to strengthen conclusions regarding CHEPs as a diagnostic tool for PSPS. Second, the potential presence of subclinical polyneuropathy in the control group cannot be entirely ruled out. Despite careful screening, undetected small fiber neuropathy may have influenced CHEP parameters. Future studies should incorporate additional assessments, such as skin biopsies or QST, to ensure a more precise evaluation of control participants. Additionally, while CHEPs proved useful in assessing spinothalamic tract dysfunction and central sensitization in PSPS, this technique does not fully capture the complexity of PSPS as a "mixed pain" condition. PSPS often involves both neuropathic and nociceptive pain components, which can be difficult to separate in the diagnostic process. Since CHEPs primarily evaluate NP mechanisms, they may not provide a complete assessment of nociceptive contributions. Future studies should consider integrating CHEPs with other diagnostic tools, such as QST or functional imaging, to better distinguish between these pain components and refine the diagnostic approach for PSPS. Finally, while this study demonstrates significant differences in CHEP parameters between PSPS patients and controls, the cross-sectional design precludes the ability to assess disease progression or treatment response over time. Longitudinal studies are needed to explore the utility of CHEPs as a monitoring tool and to determine its role in guiding therapeutic interventions for PSPS. Despite these limitations, the findings provide a strong foundation for future research, emphasizing the need for expanded studies to refine the clinical application of CHEPs in PSPS and chronic pain syndromes.

## Conclusions

This study underscores the utility of CHEPs as a non-invasive tool for assessing central pain mechanisms in patients with PSPS type 2. Significant differences in CHEP parameters, including prolonged N2 and P2 latencies and reduced N2-P2 amplitudes, were identified between PSPS patients and healthy controls. These findings suggest altered central pain processing and potential spinothalamic tract dysfunction in PSPS, supporting the hypothesis of central sensitization as a contributing factor to the condition.

Although CHEPs demonstrated diagnostic value, with N2 latency showing the highest sensitivity and specificity, limitations such as the small sample size and possible confounding factors highlight the need for further research. Future studies with larger cohorts and comprehensive analyses are essential to complement these findings and explore the clinical implications of CHEPs in diagnosing and managing PSPS. An enhanced understanding of central pain mechanisms could pave the way for targeted therapeutic strategies, improving outcomes for patients with PSPS and similar chronic pain conditions.
